# From a gene-centric to whole-proteome view of differentiation of T helper cell subsets

**DOI:** 10.1093/bfgp/elt033

**Published:** 2013-10-06

**Authors:** Tapio Lönnberg, Zhi Chen, Riitta Lahesmaa

**Keywords:** T helper cell, systems biology, proteomics, mass spectrometry, transcriptomics, T cell activation

## Abstract

Proper differentiation of naïve T helper cells into functionally distinct subsets is of critical importance to human health. Consequently, the process is tightly controlled by a complex intracellular signalling network. To dissect the regulatory principles of this network, immunologists have early on embraced system-wide transcriptomics tools, leading to identification of large panels of potential regulatory factors. In contrast, the use of proteomics approaches in T helper cell research has been notably rare, and to this date relatively few high-throughput datasets have been reported. Here, we discuss the importance of such research and envision the possibilities afforded by mass spectrometry-based proteomics in the near future.

## INTRODUCTION

**Figure 1:**
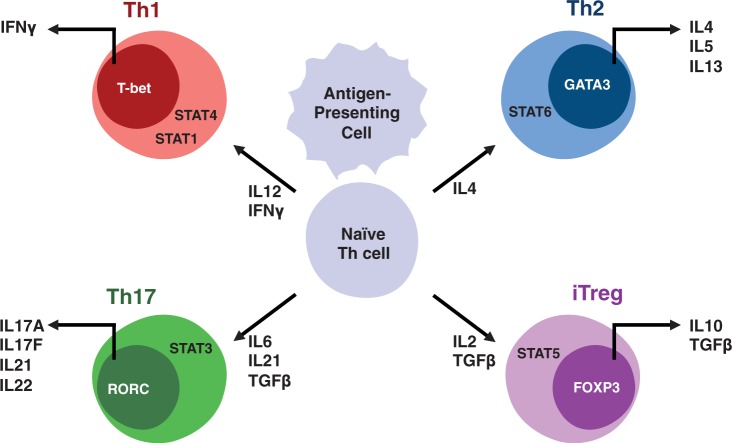
A simplistic model of the subset differentiation of T helper cells. The differentiation is initiated by contact with cognate antigen peptide–MHC complex on the antigen presenting cell. The direction of differentiation is largely determined by cytokines, the effects of which are relayed by specific STAT proteins. The STATs, together with downstream lineage-specifying transcription factors, orchestrate subset-specific gene expression programs, including production of hallmark cytokines.

CD4+ T helper (Th) cells form a vital regulatory component of the adaptive immune system. Their functional diversity is reflected in the number of alternative CD4+ T cell subsets arising from a common naïve T cell progenitor. Currently known subset repertoire includes Th1, Th2, Th17 and iTreg cells, along with several recently discovered and less thoroughly characterized subsets, such as Tfh, Th9 and Th22 cells. The differentiation and functional properties of Th subsets have been subjects of several recent comprehensive reviews [1–4]. Th cell differentiation is initiated by engagement of the T cell receptor and binding of costimulatory ligands and regulated primarily by the local cytokine microenvironment. Cytokine signals are mediated by Signal Transducer and Activator of Transcription family of proteins, which together with T cell receptor signaling activate subset-characteristic transcription factors, most importantly T-bet, Gata3, RORc and Foxp3, promoting the development of Th1, Th2, Th17 and iTreg cells, respectively (Figure 1).

Importantly, deviant Th cell activity is implicated in numerous autoimmune and allergic diseases, prompting research into the molecular underpinnings of Th effector subset development [5]. This research has relied heavily on the use of transcriptomics methods, namely DNA microarrays and more recently next-generation sequencing (NGS). In contrast, studies of Th differentiation using proteomics approaches have been relatively scarce. System-wide protein analysis has remained technically challenging due to the biochemically complex and dynamic nature of proteomes. Unlike for nucleic acids, there are no enzymatic amplification methods for peptides or proteins, leading to a need for relatively large sample amounts. Moreover, proteins are produced over a wide range of abundances (from dozens to millions of copies per cell), and are subject to numerous variations including alternative isoforms, proteolytic cleaveage and post-translational modifications (PTMs). Consequently, proteomics studies have required highly specialized instrumentation, data analysis pipelines and technical expertise. Availability of such resources has further limited the adoption of proteomics technology by immunologists. Here, we review the findings from recent studies where proteomics approaches have been applied to elucidate the processes involved in Th subset differentiation. In addition, we discuss the current limitations of such approaches and aim at foreseeing imminent development in this field. For a more thorough introduction to technical aspects of proteomics, the reader is referred to recent excellent reviews [6–9].

## TRANSCRIPTIONAL REGULATORY MECHANISMS LEADING TO SUBSET-SPECIFIC GENE EXPRESSION

Early adoption of the Affymetrix technology by two groups rapidly led to identification of new players involved in Th1 and Th2 differentiation [10–14]. Follow-up of these profiling studies have revealed, for example, the functional roles of ATF3, SATB1, PRELI and PIM kinases in human Th cell differentiation [15–18]. More recently, detailed kinetics of gene expression at early stages of mouse and human Th17 differentiation was studied by genome-wide microarrays [19, 20]. In addition, genome-wide studies on mice deficient for STAT4 and STAT6 clearly demonstrated their essential role in directing Th1 and Th2 differentiation [12, 13, 21, 22]. Altogether, these studies lead to identification of hundreds of genes expressed differentially in response to cytokine-STAT signalling [23]. To further distinguish which genes are direct targets of STATs, chromatin immunoprecipitation (ChIP)-Seq has been used to map the binding sites of STAT4, STAT6, STAT3 and STAT5 in response to IL-12, IL-4, IL-6 and/or IL-21, IL-2 stimulation using both wild type and STAT-deficient T cells [21, 22, 24–28]. Although it is well established that the STATs are important for cytokine signalling and initiation of differentiation processes, these studies established that even in differentiated Th1, Th2, Th17 or iTreg cells, numerous STAT binding sites were mapped to the signature cytokine loci. ChIP has also been applied to identify targets of the hallmark transcription factors T-bet and GATA3 in Th1 and Th2 cells. Interestingly, a set of genes including *IL4* and *IFNG,* is bound by GATA3 in both Th1 and Th2 cells and also targeted by T-bet in Th1 cells, supporting their opposite regulatory roles in Th1/Th2 fate decision [29].

However, identification of binding sites of transcription factors does not alone indicate functional impact of such binding. To address this question, further maps of transcription factor binding have been combined with detection of chromatin modification markers such as histone H3 lysine 4 (H3K4) and lysine 27 (H3K27) trimethylation, enriched in promoters or repressors, respectively, as well as expression levels of neighbouring genes [21, 30]. For example, H3K4me3 and STAT4, STAT6 or STAT3 are found to be associated with *Ifng*, *Il4* or *Il17*, respectively, in differentiated Th1, Th2 and Th17 lineages [21, 22, 24]. Interestingly, STAT proteins appear to induce gene expression in one lineage and inhibition of associated active epigenetic marks in another lineage. In the case of GATA3 and T-bet, these factors seem to regulate both expression of Th2 or Th1 lineage-specific genes as well as their active and repressive histone marks [30].

Furthermore, active enhancer landscapes have been mapped in Th1 and Th2 cells [31, 32]. Consensus motifs for STAT1 and STAT4 are enriched in Th1 enhancers, whereas STAT6 motifs are found in Th2 but not Th1 enhancers. Binding of these STATs can activate lineage-specific enhancers and suppress enhancers associated with alternative cell fates [31].

To get an insight into human diseases it is necessary to carry out studies in primary human cells in addition to mouse studies. Genome-wide association studies (GWAS) have identified a large number of single-nucleotide polymorphisms (SNPs) that are associated with autoimmune diseases. The majority of these SNPs are, however, in the non-coding regions of DNA. To understand how these SNPs might contribute to disease pathogenesis, it is crucial to find out what is the function of such regions of non-coding DNA. A recent integrative study on human cells that included analysis of chromatin state maps, transcription factor-binding sites and modelling of enhancer-gene pairs combined with GWAS studies of autoimmunity-associated SNPs suggest a direct role for distal regulatory elements in disease aetiology. Such SNPs were shown to alter binding of transcription factors involved in Th cell differentiation [32]. Extension of such studies that map the landscape of regulatory SNPs in human T cell biology will be vital in order to better dissect and understand the molecular mechanisms of immune-mediated diseases in man.

## FROM TRANSCRIPTOMES TO SUBSET-SPECIFIC PROTEOMES

Transcriptional control mechanisms only account for part of the regulation of gene expression. In fact, only ∼40% of variation in protein quantities can be explained by variation in mRNA quantities [33]. Thus, for a conclusive protein-level view, direct proteomics analysis is required. Differentially expressed proteins can be identified by direct mass spectrometric analysis after sufficient fractionation, or based on visual comparison of two-dimensional electrophoresis (2DE) patterns. Quantification can be performed using isobaric labelling or so-called label-free approaches a relying on careful alignment of extracted ion chromatograms [7].

However, while current state-of-the art transcriptomics afford complete system-wide detection of analytes, proteomic techniques have only recently begun to approach comparable level of coverage. Thus, the detection of proteins is typically limited and biased towards the higher abundance molecules. Accordingly, relatively few proteins have been identified as differentially regulated in response to Th-polarizing cytokines. Furthermore, such studies have often failed to recapitulate known expression patterns of subset-specific hallmark molecules, such as transcription factors and cytokine receptors, often present at low copy numbers (100–1000 copies per cell) [34].

**Figure 2:**
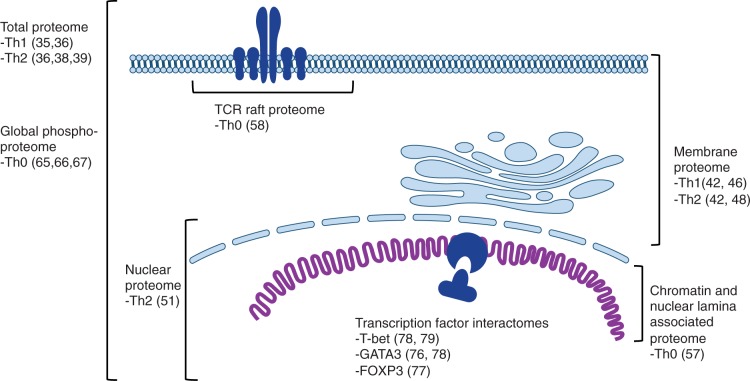
Proteomics approaches to elucidate processes leading to the development of T helper effector subsets. Th0, Th1 and Th2 refer to experimental conditions where cells were activated (Th0) and cultured in presence or absence of cytokines promoting differentiation of Th1 or Th2 cells.

The global effects of IL12 signalling have been investigated in both early (24 h) and late stages (7 and 14 days) of human Th1 differentiation (Figure 2) [35, 36]. The lists of IL12-regulated proteins in these different time points showed little overlap, likely resulting from incomplete coverage achieved in each study as well as the temporally dynamic nature of Th subset differentiation. For example, Cyclophilin A was detected as upregulated in Th1-promoting conditions at Day 1 but no longer at Days 7 or 14. Nevertheless, both studies were successful in detecting known Th1-associated proteins. At 24 h, programmed cell death 4 (PDCD4), a known IL12 target, was measured as 2.5-fold induced. At the later time points, the IL12-induced proteins were enriched for known targets of interferons, including interferon-induced protein 35 (IFP35), ISG15 ubiquitin-like modifier (UCRP) and Tryptophanol-tRNA synthetase. In fact, several of these proteins had been earlier identified as direct targets of interferon-α in a similar experimental model [37]. These findings illustrate the importance of positive feedback delivered by autocrine and paracrine signalling during Th differentiation.

Proteomic responses to the Th2-promoting IL4 signalling have been studied both in human [36, 38] and by using Stat6-/- mice [39]. As with IL12, the resulting data were of limited coverage and contained many structural or otherwise highly abundant proteins. In the context of Th2 differentiation, the most interesting observations were upregulation of core binding factor subunit beta, isoform 2 (CBFb2) and cellular nucleic acid binding protein (CNBP). Both proteins are implicated in regulation of gene expression. CNBP can regulate gene expression at both transcriptional and translational levels. CBFb2, in turn, is a co-factor for Runx family transcription factors. Notably, RUNX1 is an inhibitor of GATA3 expression and a direct target of STAT6 [28, 40]. Furthermore, the RUNX1–CBFb complex has been shown to be necessary for Treg development [41].

The above mentioned results were obtained by the use of 2DE, which is limited in its ability to resolve poorly soluble hydrophobic membrane proteins. In a complementary study by Loyet *et al.* [42], plasma membrane preparations from human Th1 and Th2 cells were compared (at 7 days post-activation). The authors identified bone marrow stromal protein 2 (BST2) and T cell receptor interacting molecule (TRIM), among the proteins most upregulated in Th1 conditions. BST2, also known as tetherin, plays a role in lateral organization of plasma membrane microdomains [43]. TRIM is associated with some, but not all T cell receptor complexes and plays a role in thymic selection [44, 45]. The exact role of these proteins in Th differentiation remains to be elucidated. By targeting membrane-derived microsomal fractions, Filén *et al.* [46] found two physically interacting proteins, galectin-1 and CD7, to be downregulated by IL12. Galectin-1 has since been reported to be produced by Th2 cells, promoting production of Th2 cytokines and antagonizing Th1 survival [47]. Conversely, in response to IL4, GTPase of the immunity-associated protein 4 (GIMAP4) was found to be downregulated [48]. Furthermore, transcriptomics analysis revealed that GIMAP4 is upregulated in Th1 cells. Members of the GIMAP family are expressed in specific patters during T cell development, with expression of GIMAPS 3, 4, 5 and 7, being increased during the transition from the double positive to the single positive stage. However, deletion of GIMAP4 does not impair T cell generation, but instead seems to be involved in caspase-3-mediated apoptosis [49, 50]. Importantly, GIMAP4 seems to regulate cytokine production by Th cells (Heinonen *et al.*, unpublished observation).

Besides absolute abundance, protein activity can be regulated by differential localization in subcellular compartments. Targeted studies of subcellular fractions can thus provide biologically meaningful information beyond the context of global gene expression. In particular, transcription factors are frequently inducibly recruited to or retained in the nucleus in response to specific signals. During Th2 differentiation, phosphorylated STAT6 localizes preferentially to the nucleus, leading to higher relative increase in concentration than suggested by the IL4-induced increase in the level of the STAT6 mRNA. To identify other IL4-regulated proteins potentially involved in transcriptional or epigenetic regulation, nuclear fractions of human Th2 cells have been analysed using iTRAQ-based quantitative proteomics [51]. The resulting list of 30 proteins, included two IL4-induced transcription factors, Y box binding protein 1 (YBX1) and Ikaros (IKZF1), not belonging to the canonical Th2 or Th1 pathways, although the latter has been recently shown to directly inhibit expression of T-bet and activate expression of Th2 cytokines [52, 53].

In some of these studies, differential expression was also investigated at the RNA level using either microarray datasets or quantitative RT–PCR. Rautajoki *et al.* [36] observed a correlation between protein abundance changes and microarray data in 6 out of 11 cases. Differential regulation of membrane-associated BST2 and TRIM was not observed at the mRNA level [42]. In the study of IL4-responsive nuclear proteins by Moulder *et al.* [51], significant transcriptional regulation was detected in three out of eight cases. Altogether, such comparisons are complicated by considerations of subcellular localization, extracellular secretion, temporal separation of transcription and translation and uncertainty associated with assignment of peptides to alternative isoforms. In mammalian cells, protein abundance has been shown to be predominantly regulated at the level of translation. Protein copy numbers correlate strongly with the rate of translation, with proteins of highest abundance translated at least 100 times more efficiently than the least abundant proteins [34, 54].

Translation efficiency is in part regulated by eukaryotic translation initiation factors (eIF). Interestingly, eIF4E, regulator of mRNAs involved in processes including cell cycle, innate immunity and apoptosis, is expressed at a higher level in activated Foxp3- than Foxp3+ T cells. Furthermore, inhibition of eIF4E activity lead to upregulation of Foxp3 [55]. It seems plausible that similar post-transcriptional control is also associated with other Th subset differentiation programs.

## CONTRIBUTION OF T CELL ACTIVATION TO PROTEIN EXPRESSION AND LOCALIZATION DURING SUBSET DIFFERENTIATION

Significant changes of gene expression occur in response to T cell activation through CD3/T cell receptor (TCR) complex and costimulatory receptors, correlating with initiation of cell cycle progression, growth and metabolic changes. As naive T cells are generally quiescent and arrested at G0, they lack a number of proteins required for entry into and completion of cell cycle [56, 57].

In an extensive recent work by Orr *et al.* [57], changes associated with activation and cell cycle initiation were investigated both on a systemic level as well as in terms of proteins specifically recruited to chromatin or to the nuclear lamina. Among the total of 2894 proteins identified, 1724 were associated with the chromatin fraction. Notably, the entry to G1 stage of cell cycle was accompanied with a significant shift in protein expression, with the abundance of 772 proteins increased and 630 decreased. Of the chromatin or nuclear matrix-bound proteins, 307 were increased and 211 decreased. Many of the induced proteins were involved in ribosome biogenesis, reflecting the acceleration of protein synthesis and growth during cell cycle entry. Importantly, by silencing expression of splicing factor 3b, subunit 2 (SF3B2) and eIF6 the authors showed that cell cycle progression is not obligatorily connected to cellular growth. Furthermore, contrary to previous knowledge, the data indicated that xin actin-binding repeat containing 1 (XIRP1) protein is expressed in human T cells and inducibly associated with the chromatin fraction. The role of this relatively poorly characterized protein in T cell activation is not known. In addition to chromatin, the local microenvironment of the T cell receptor is of particular interest in the context of T cell activation. The proteome of this fraction was characterized in a recent targeted study by de Wet *et al.* [58]. The authors identified proteins associated with the formation of the immunological synapse, endocytosis of the TCR as well as negative regulators of activation.

Importantly, full activation of T cells requires engagement of the T cell receptor and concomitant signalling through the costimulatory receptors, in particular, CD28. The pathways activated by the two stimuli are partially overlapping, both leading to activation of NFκB and NFAT [59]. The relative contribution of CD28 signalling to proteomic changes following T cell activation has been studied using 2DE. Kronfeld *et al.* [60] have identified 35 proteins regulated in response to either of two CD28 ligands, B7-1 or B7-2. These proteins were, in general associated with activation- and proliferation-related processes. More recently, Lichtenfels *et al.* [61] have identified a panel of 52 proteins regulated by CD3, CD28 and the downstream effector, cytokine IL2. Again, the functional annotations of these proteins were in agreement with biological processes related to T cell activation, with 18 proteins annotated with metabolic processes, 15 with transport, seven with cytoskeleton organization and six with signal transduction.

Altogether, T cell activation leads to considerable reorganization of the proteome, which to a great extent reflects the alterations of cellular morphology and metabolism. Many of these of changes have been characterized in studies of relatively late points of T cell activation, i.e*.* hours or days. For insights into the early events of activation, the role of post-translational modifications has to be taken into consideration.

## EARLY SIGNAL TRANSDUCTION BY PTMs

PTMs provide rapid and reversible mechanisms for regulating protein activity. On average, each protein has been estimated to be a target for 2.5 modifications [62]. In particular, phosphorylation of tyrosine, serine and threonine residues are widely used as means of signal transduction. Proteomics has the unique capacity to identify new PTM sites and quantify PTMs in a system-wide fashion.

The main challenges for studies of PTMs, including phosphorylation, arise from the typically low ratio of the modified protein or peptide and the unmodified form. Recently, several mass spectrometry-compatible system-wide strategies allowing selective isolation of phosphorylated proteins or peptides have been developed [63]. These include immobilized metal affinity chromatography (IMAC), titanium dioxide resins and immunoprecipitation with antibodies against phosphorylated amino acid residues, in particular, phosphotyrosine (pY). With these tools, more than 100-fold enrichment can be achieved [7 and references 93–95 therein]. The methods can also be used in combination. For example, the flow through fraction from TiO_2_ affinity chromatography can be further precipitated by anti-pY antibodies.

Using global phosphopeptide enrichment strategies, thousands of phosphosites can be detected. However, large amounts of sample material are consumed in such analyses. Therefore, most T cell-related phosphoproteomics studies have been performed using the Jurkat T cell line [64]. In activated Jurkat cells, more than 10 000 phosphorylation sites have been identified [65]. With more limited material from primary CD4+ T cells, 2800 sites have been identified [66]. The Majority of the identified sites are in serine residues, followed by threonine and tyrosine at an approximate ratio of 88, 10 and 2%, respectively [67]. Despite the relative scarcity of phosphotyrosine sites, they are regarded as particularly important, as they have well-documented roles in regulating protein activity through both conformational change and creating specific binding sites for Src homology 2 (SH2) domains found in a diverse set of proteins involved in signal transduction [68]. Importantly, both proximal T cell receptor signalling as well as cytokine-responsive JAK-STAT pathways rely on successive tyrosine phosphorylation events. Consequently, a number of studies have concentrated exclusively on the role of tyrosine phosphorylation in T cell signalling [69].

In several studies, quantitative changes in the phosphoproteome in response to T cell activation were measured. These studies have generally focused on the early time points following TCR engagement, as it has been shown that cellular phosphotyrosine levels reach their maximum level already at 1–2 min after activation, and phosphoserine and phosphothreonine at ∼20 min [66]. In the studies with the highest coverage so far, 696 phosphorylation sites on 453 distinct proteins were found responsive to T cell receptor stimulation [65]. These included many novel phosphorylation sites on proteins with known roles in T cell activation as well as new sites on proteins previously not implicated in T cell activation. Such findings, together with the limited overlap of phosphosites identified between studies suggest that the full complexity of T cell phosphoproteome has not yet been reached by current investigations.

T cell responsive phosphoproteins are found in all major cellular compartments, although they are particularly enriched in the plasma membrane [66]. Similarly, TCR-regulated phosphorylation events pervade all cellular processes associated with T cell activation. The largest group of these sites is related to activation-induced morphological changes, such as formation of immunological synapse and patterning of T cell surface proteins. Interestingly, proteins annotated with transcriptional regulatory functions seem in general to be dephosphorylated in response to activation [66].

Global phosphoproteome responses to key cytokines promoting differentiation of Th subsets have not been characterized. Recently, Osinalde *et al.* applied phosphotyrosine immunoprecipitation on cells of the Kit225 T cell line, stimulated for 5 min with IL2 [70]. The authors reported 56 proteins reproducibly enriched in the immunoprecipitated fraction. For 33 of these proteins, specific phosphotyrosine sites could be identified, other proteins being likely to co-precipitate due to protein–protein interactions with the phosphorylated proteins. Accordingly, the identified proteins were enriched for SH2 domains. Functionally, the identified proteins were enriched for categories including cellular growth and proliferation well in line with the established functions of IL2 and the IL2-dependent nature of the Kit225 cell line.

In contrast with immortalized cell lines, studies of cytokine signalling using primary Th cells in general require additional stimulation of the TCR, as T cell activation is necessary for cytokine-induced subset differentiation as well as for expression of receptors for several cytokines including IL2. However, as T cell activation leads to widespread phosphorylation of abundant proteins, an important challenge will be detecting the events induced specifically by cytokine signalling, and determining the extent of cross-talk between the T cell activation and cytokine-induced pathways.

An important trend emerging from the findings of such system-wide studies is the close relationship of protein phosphorylation and protein–protein interactions. While binding of phosphorylated tyrosine residues with SH2 domains has been recognized for long, similar mechanisms seem to frequently play roles also in serine and threonine phosphorylation. More specifically, it has been shown that threonine and serine residues phosphorylated in response to TCR activation are enriched in protein domains that are part of protein–protein interaction surfaces [65]. Activation-induced phosphorylation can influence formation of interaction either negatively or positively. For example, expression of Fos and Il2 is promoted by serine phosphorylation-induced disruption of interactions between co-repressors nuclear receptor co-repressor 2 (NCOR2), Brahma protein homolog (BRG1), and heterochromatin protein 1 homolog (HP1α) and their respective transcription factors. In contrast, patterning of T cell surface molecules associated with T cell activation is dependent on phosphorylation-induced interactions [65].

Finally, it is becoming increasingly evident that phosphorylation is not the only form of PTMs involved in T cell signalling. Activity of STAT proteins is, in addition to phosphorylation, controlled by lysine acetylation [71]. Arginine methylation has been reported to be involved in processes controlling expression of Th2 cytokine locus [72]. While antibody-based methods for global enrichment of both acetylated and methylated peptides exist, up to this date, they have not been applied in T cell research [73]. Importantly, the challenges associated with PTM mapping are multiplied when combinatorial effects of modifications are taken into account [74].

## REGULATION OF LINEAGE-SPECIFYING TRANSCRIPTION FACTORS BY PROTEIN–PROTEIN INTERACTIONS

Functional activity of proteins involves transient protein–protein interactions and formation of stable protein complexes. With affinity purification and mass spectrometry, binding partners of a protein can be interrogated in an untargeted fashion, offering important insight to protein function [75]. Currently, the ultimate goal of interactome mapping, cataloguing the full network of protein–protein interactions, remains to be completed in mammalian cells. Furthermore, protein–protein interactions are subject to context-specific regulation. A pair of proteins can therefore, in different conditions, exhibit varying tendency to interact due to changes in regulatory signals or stoichiometric ratios of binding partners. Therefore, it is crucial to study protein–protein interactions in specific biological systems.

In the context of Th cell differentiation, of key importance are the interactions involving the lineage-specifying transcription factors, as their DNA binding capacity and regulatory potential can be modified by a variety of co-factors. For example, GATA3 has been reported to form two distinct complexes, GATA3/CHD4/p300 and GATA3/CHD4/NURD [76]. The former is found at Th2 cytokine loci and induces transcription, whereas the latter is repressive and found at Tbx21 locus. Furthermore, the lineage-specifying Treg transcription factor FOXP3 has been identified as part of large (400–2000 kDa) protein complexes, with more than 300 potential binding partners [77]. Notably, FOXP3 directly regulates the expression of many of these co-factors. The opposing regulatory roles of some co-factors suggest that FOXP3 can form functionally distinct complexes in response to specific cues. This finding provides a potential mechanistic explanation for the functional heterogeneity associated with Treg cells.

Interestingly, the lineage-specifying transcription factors can also in many cases interact with each other. GATA3 interacts with tyrosine-phosphorylated T-bet, negatively influencing its binding to DNA and thus antagonizing Th1 differentiation [78]. T-bet, in turn, is able to interact with the Tfh-promoting BCL6 and coordinately regulate gene expression [79]. With FOXP3, remarkably many of the identified interacting proteins are transcriptional regulators implicated in Th cell differentiation, including GATA3, RUNX1, NFAT, STAT3 and IKZF1 [77]. Altogether, it is turning out that the lineage-specifying transcription factors form a densely connected regulatory network. The physical interactions between the nodes of this network are likely to provide an important component in regulation of Th cell subset differentiation and plasticity [80]. More detailed elucidation of kinetics of such interactions in response to TCR and cytokine stimulation could provide further insight into these processes.

## FUTURE OUTLOOK

Proteomics is a field in rapid development and in many ways its full potential remains to be exploited. In particular, studies with limited sample material, e.g. from primary cell cultures, have suffered from the restricted sensitivity and dynamic range of mass spectrometry-based approaches.

In the context of Th cell differentiation, many of the key proteins, such as transcription factors, kinases and regulators of calcium signalling or cell adhesion are found at low copy numbers (<5000 copies per cell) [81]. Proteome level investigation of many biologically crucial processes would thus require reproducible detection and quantification of such low abundance proteins. In addition, improvements in sensitivity would enable analyses with smaller amounts of sample material. For example, in the case of primary cells, the sample could potentially be analysed as biologically meaningful subpopulations defined by expression of surface markers. Notably, heterogeneity within Th cell responses is becoming increasingly appreciated, and studying the Th cell subpopulation structures with a greater resolution could in many cases lead to greater biological insight.

The ultimate goal for analytical sensitivity is the ability to study proteomes derived from single cells. Such analyses would take into account the ubiquitous cellular heterogeneity and describe nature of responses to external stimuli more accurately than studies relying solely on population-level averages. Although the important milestone of single-cell resolution has recently been reached by transcriptomics, the challenges involved in a similar proteomics strategy are substantial. All current single-cell transcriptomics approaches require significant amplification of starting material—something not possible at the protein level. Although each mRNA molecule on the average gives rise to 2800 proteins [54], the single cell proteomics analysis would still be performed with a very limited number of molecules. Furthermore, cell lysis, digestion and sample fractionation would have to be extremely efficient and reproducible. To date, single-cell mass spectrometry experiments have only been reported with highly abundant peptides, lipids or metabolites [82]. Arguably, the most powerful tools for high-throughput single-cell protein-level investigation are currently offered by multi-parameter flow cytometry and the recently developed mass cytometry [83]. However, these approaches are based on the use of antibodies with all the associated theoretical and practical limitations.

Importantly, detection sensitivity is closely related to the concept of dynamic range. The concentrations of cellular proteins vary over seven orders of magnitude [54, 81, 84]. Thus, a pool of tryptic peptides from a complete cellular proteome will be dominated by peptides from the highly abundant proteins. A mass spectrometer operating in a typical data-dependent acquisition mode is thus prone to repeatedly sampling ions from relatively few common proteins, and missing the more rare ions entering the instrument simultaneously. The problem has been partially alleviated by the faster duty cycles, i.e. sampling times used in modern instruments. In addition, sample can be subjected to extensive chromatographic fractionation prior to analysis, which however can also lead to sample loss [85]. Alternatively, with prior information of the sample, assays can be designed, which target and quantify specific peptides of interest. With these techniques, generally called selective reaction monitoring (SRM) or multiple reaction monitoring (MRM), individual peptides are selected and fragmented, typically using a triple quadrupole instrument and resulting fragment ions of expected mass to charge ratio (referred to as transitions) are quantified [86]. In yeast, the dynamic range of a single SRM-based analysis has been demonstrated to span six orders of magnitude, from 1.3 million to <50 copies per cell [87]. However, whereas high sensitivity and dynamic range can be achieved, designing and validating the assays do require considerable efforts. Recently, a conceptually related global analysis strategy, SWATH-MS, was introduced. With this data-independent acquisition-based strategy, dynamic range of four orders of magnitude was reached [88].

Besides improvements in sensitivity, studies of Th cell biology would benefit from straightforward methods for absolute protein quantification. Isobaric peptide labels are typically used to determine relative quantities of a peptide or protein between two samples with no information gained on abundance differences between protein species. According to recent reports, Th cell lineage specification is likely to result from quantitative effects of opposing transcription factors [80, 89, 90]. Moreover, concentration of a transcription factor is typically correlated with DNA occupancy. Thus, determination of protein copy numbers per cell would enhance explanatory power of studies targeting regulatory networks leading to Th differentiation. Although strategies for absolute quantification using either spike in peptides [91] or bioinformatics analysis [92] have been developed, neither are yet routinely used. Notably, in a recent investigation Simicevic *et al.* [93] used SRM-based workflow to determine absolute quantities of 10 key transcription factors involved in adipocyte differentiation, revealing that the copy numbers vary considerably, from ∼250 to over 300 000 molecules per cell [93]. Similar approaches would likely prove to be informative also in the case of T cells.

In conclusion, several features of Th cell differentiation remain to be elucidated at the protein level. These include the regulatory network of lineage-specifying transcription factors, in particular, determination of absolute copy numbers, post-translational modifications, mutual interactions and temporal regulation in subset polarizing conditions. The rapid technical development of mass spectrometry together with the increasing rate of adoption of proteomics methods among immunologists offer promise that many of these questions can soon be addressed.

**Key Points**

- Although transcriptional regulation underlying differentiation of Th cell subsets has been intensively studied, there are relatively few studies on the proteome level.
- The investigations of subset-specific protein expression have, to this date been limited by analytical sensitivity and dynamic range. Nevertheless, panels of proteins expressed with subset-specific patterns have been successfully identified. Expression of these proteins does not always correlate with the expression of respective mRNAs.
- Global analyses have revealed extensive reorganization of the proteome in response to T cell activation. This reorganization takes place at the levels of protein expression, localization and phosphorylation, and correlates with the formation of the immunological synapse and the initiation of cellular proliferation.
- Recent reports suggest that the key lineage specifying transcription factors in many cases have the potential for physical interaction. Th cell research would benefit from systematic characterization of such interactions and their temporal kinetics during subset differentiation.

## FUNDING

The Academy of Finland Decision 258313, European Union FP7 Grant ‘Systems Biology of T-cell activation in health and disease’ (SYBILLA); The Academy of Finland (Centre of Excellence in Molecular Systems Immunology and Physiology Research, 2012–2017, Decision 250114); The Sigrid Jusélius Foundation; The National Technology Agency of Finland (TEKES).
